# Prevalence of high frequency hearing loss consistent with noise exposure among people working with sound systems and general population in Brazil: A cross-sectional study

**DOI:** 10.1186/1471-2458-8-151

**Published:** 2008-05-07

**Authors:** Regina P El Dib, Edina MK Silva, José F Morais, Virgínia FM Trevisani

**Affiliations:** 1 Urgency Medicine and Evidence-Based Medicine Department, the Brazilian Cochrane Centre, the Federal University of São Paulo, Brazil; 2 Actuary and Quantitative Methods Department, Pontifical Catholic University of São Paulo, Brazil

## Abstract

**Background:**

Music is ever present in our daily lives, establishing a link between humans and the arts through the senses and pleasure. Sound technicians are the link between musicians and audiences or consumers. Recently, general concern has arisen regarding occurrences of hearing loss induced by noise from excessively amplified sound-producing activities within leisure and professional environments. Sound technicians' activities expose them to the risk of hearing loss, and consequently put at risk their quality of life, the quality of the musical product and consumers' hearing. The aim of this study was to measure the prevalence of high frequency hearing loss consistent with noise exposure among sound technicians in Brazil and compare this with a control group without occupational noise exposure.

**Methods:**

This was a cross-sectional study comparing 177 participants in two groups: 82 sound technicians and 95 controls (non-sound technicians). A questionnaire on music listening habits and associated complaints was applied, and data were gathered regarding the professionals' numbers of working hours per day and both groups' hearing complaint and presence of tinnitus. The participants' ear canals were visually inspected using an otoscope. Hearing assessments were performed (tonal and speech audiometry) using a portable digital AD 229 E audiometer funded by FAPESP.

**Results:**

There was no statistically significant difference between the sound technicians and controls regarding age and gender. Thus, the study sample was homogenous and would be unlikely to lead to bias in the results. A statistically significant difference in hearing loss was observed between the groups: 50% among the sound technicians and 10.5% among the controls. The difference could be addressed to high sound levels.

**Conclusion:**

The sound technicians presented a higher prevalence of high frequency hearing loss consistent with noise exposure than did the general population, although the possibility of residual confounding due to unmeasured factors such as socioeconomic status cannot be ruled out.

## Background

Music is ever present in our daily lives, establishing a link between human beings and the arts through the senses and pleasure. However, if we use music inappropriately, that is, if we make abusive use of the duration, intensity and/or frequency of exposure to music, the noise may have an adverse impact on our hearing function.

High frequency of hearing loss consistent with noise exposure (noise-induced hearing loss, NIHL), excluding cases of acute acoustic trauma, is characterized by the gradual and progressive loss of hearing acuity. NIHL occupies the second place among the most common hearing illnesses, only surpassed by presbyacusis [1,2].

In Brazil, noise has only been recognized as an ambient pollutant since March 1991 [3]. Epidemiological data regarding hearing loss in Brazil is scarce and only relates to certain fields of activity. Thus, there are no epidemiological records characterizing the real overall situation. The data on the prevalence of hearing loss that are available provide a partial picture of the risk associated with this condition.

It has been estimated that 25% of the worker population exposed to noise have some degree of NIHL. In spite of being the most frequent type of damage to workers' health, the prevalence of NIHL among Brazilian workers is still unknown [4].

In the United States, approximately ten million workers have NIHL > 25 decibels (dB) [5]. In the United Kingdom, NIHL was the second most frequent workplace-related illness officially recorded in 1990 and 1991 [6].

According to data from the World Health Organization (WHO), approximately 16% of the world's population endures NIHL caused by occupational exposure to noise [5].

NIHL is generally sensorineural, bilateral, symmetrical and irreversible. Individuals with this illness are likely to present a prolonged history of exposure to high noise levels (> 85 dB(A) for eight hours a day), sufficient to have caused a loss in the levels and configurations observed in audiological tests. Hearing loss consistent with noise exposure is likely to have gradually developed over a period of between 5 and 20 years of general exposure in all locations [7,8].

Recently, there has been rising concern about the prevalence of NIHL caused by excessively amplified sound activities in leisure settings. Among these activities, the exposure of musicians and sound technicians (professionals involved in sound mixing, microphone operation and video material editing) who are exposed to intense and long lasting music have been highlighted.

With regard to sound technicians who begin their careers early in life and consequently are frequently exposed to high-intensity sounds, it needs to be asked how the hearing levels of these individuals are holding up, when music is the medium through which they perform their professional activities.

Sound technicians are the agents who transport to consumers one of Brazil's best products: Brazilian popular music. Dealing with and watching over what this process entails is of great social relevance to the country's health, culture and economy.

Moreover, decreases in hearing function worsen individuals' quality of life, communication abilities, educational achievements and enjoyment of leisure activities, and have enduring economic consequences [9].

Thus, the present study had the aim of gauging the prevalence of high prevalence hearing loss consistent with noise exposure among sound technicians and comparing this with a control group without occupational noise exposure.

## Methods

This study was approved by the Ethics Committee of the Federal University of São Paulo. It was a cross-sectional comparative study between two groups: sound technicians and individuals without occupational noise exposure (controls). The sample consisted of 177 participants: 82 people working with sound systems and a sample of 95 members of the general population (control group). The inclusion criterion was that participants needed to be adults > 19 years old.

The sound technician group included professionals involved in sound mixing, audio equipment operation, video material editing, video equipment operation and microphone operation, working in closed or open spaces, with at least five years of exposure to sound-producing activities [7,8].

The control group consisted of individuals without a history of exposure to continuous noise, i.e. individuals who were rarely exposed to music and whose work activities did not involve music. Workers exposed to high levels of industrial noise, such as factory workers, bus drivers and the like, were also excluded from this group.

The following were exclusion criteria for both the sound technician and control groups: use of individual sound amplification devices (hearing aids); diagnosis of congenital hearing deficiency (genetic or acquired); reduced external acoustic ear canal; and presence of excess earwax or otorrhea.

Using the criteria outlined above, we excluded 19 subjects from the sound technician group because 11 of them had been exposed to sound-producing activities for less than five years and eight subjects were less than 19 years old. In the control group, we excluded seven subjects: six because they did not fulfill the inclusion criterion regarding age and one because of the presence of excess earwax.

The control group was selected by invitation. The participants in this group were health professionals, students and employees at the Federal University of São Paulo, Brazil, who were unaware of the possible presence of hearing loss.

The evaluations and questionnaires were applied in the Brazilian Cochrane Center and in the studios of the television channels Record TV and Cultura TV, in São Paulo, Brazil. Portable acoustic booths were used when conducting the audiometric examinations. Rooms were deemed to be silent room when the noise level was between 55 and 60 dB, in accordance with Administrative Technical Standard no. 3214 of the Ministry of Labor and the Workplace Safety Office [10].

The sound pressure levels in the examination locations were measured using devices that conformed to the standard ANSI S1.4-1983. These made it possible to check whether the environment within which the booth had been set up was appropriate for conducting audiometric assessments.

The sound pressure levels in the places where the audiometric assessments were carried out were measured using the TES 1350A sound level meter. In the Record TV studio, the device measured a minimum of 57 dB(A). In the Cultura TV studio and in the Brazilian Cochrane Center, the estimated minima were measured as 41.6 dB(A) and 57.8 dB(A), respectively. All three locations where acoustic measurements were made were within the above mentioned standard set by the Ministry of Labor and the Workplace Safety Office [10].

Individuals who presented a diagnosis of hearing abnormalities during the examination (at any of the hearing frequencies assessed), or a diagnosis of obstruction, were referred to the outpatient clinic of the Speech-Language-Hearing Service of Hospital São Paulo, the Federal University of São Paulo, Brazil, for detailed evaluation and further procedures as required.

We investigated the participants' hearing levels at the following frequencies: 250 Hz, 500 Hz, 1000 Hz, 2000 Hz, 3000 Hz, 4000 Hz, 6000 Hz and 8000 Hz. We also performed speech audiometry [11].

The subjects were considered to present high frequency hearing loss consistent with noise exposure if their audiograms at the frequencies of 3000 and/or 4000 and/or 6000 Hz presented hearing thresholds that were at least 25 dB higher than the thresholds for the other tested frequencies (i.e. 250 Hz, 500 Hz, 1000 Hz, 2000 Hz and 8000 Hz), when this compromised hearing was observed in both the air test and the bone test, in one or both ears [4].

The sound technicians were asked to take a 14-hour hearing rest prior to the test, in accordance with Administrative Technical Standard no. 19 of the Ministry of Labor and the Workplace Safety Office, dated April 22,1998 [4]. We applied a questionnaire about music listening habits (we asked whether participants were going to discos and/or listening to walkmans or iPods at least once a week, in the past or present) and use of hearing protection devices. Participants in the sound technician group were considered to be using hearing protectors if they reported using them for at least 20% of their total time at work. For example, if an individual worked as a sound technician for nine years but used hearing protection devices for only two years (9 × 20% = 1.8 years), it would be deemed that this individual had indeed used hearing protection. We also gathered data on correlated complaints, ototoxicity exposure and length of exposure to sounds. Visual inspection of the participants' ear canals was performed using an otoscope, in order to rule out any presence of obstructions that would alter the examination results [10].

A basic tinnitus assessment was performed, including an audiological evaluation and a self-reported assessment. We also defined tinnitus as a ringing or other type of noise generated within the head [12].

The audiological evaluation was performed using the portable AD229 E digital audiometer, calibrated according to the standard ANSI S3.6 (1989) [4,13,14].

### Sample size

To determine the sample size to be used for comparing hearing loss prevalences between the sound technician group (P_PE_) and the control group (P_PNE_), pairs of ratios were compared by means of the equation proposed by Pocock (1983) [15]:

$$n=\frac{\left(P_{PNE}(1-P_{PNE})+P_{PEt}(1-P_{PE}\right)}{{\left(P_{PNE}-P_{PE}\right)}^{2}}\Theta^{2}$$

Where Θ = *Z_α _*+ *Z_β_*, such that Z_α _and Z_β _were respectively the confidence scores linked to the confidence intervals (CI) (1-α) × 100% and (1-β) × 100%.

The prevalences of hearing loss among a group of percussionists and in the general population were estimated by Cunningham (2006) [16], respectively, as P_PE _= 0.39 and P_PNE _= 0.9. In the present study, it was intended to carry out the comparison at a significance level of α = 5% with an elasticity trait of 1-β of 95% and a two-tailed hypothesis test. Therefore, the equation obtained was Θ = *Z_α _*+ *Z_β _*= 1.65 + 2.94, thereby recommending the use of at least 50 participants in each group.

### Statistical analysis

In the inferential portion of the study, we used Student's t test for homogenous independent samples, whenever the comparison variable (outcome variable) was continuous, presented normal or near-normal distribution (evaluated by the Kolmogorov-Smirnov test) and presented equal variances (evaluated by Levene's test). To compare ratios, we used the chi-square test or Fisher's exact test, in accordance with the conditions established by Cochran (1954) [17]. The tables and statistical calculations were built up in the SPSS software for Windows, version 13.0. P-values less than 0.05 were taken to be statistically significant.

## Results

The minimum sound pressure levels for each of the sound technicians' workplace environments were measured in the studios of the television channel Cultura TV, in order to exemplify how much noise the workers were exposed to. In the audio operators' studio, 101 dB(A) was detected. In the sound mixing work area, videotape operators' work area and microphone operators' work area, the measurements were respectively 91.1 dB(A), 90.7 dB(A), and 94.2 dB(A). In the videotape editing and sound monitors' areas, the measurements were 89.1 dB(A) and 86.6 dB(A), respectively.

Table 1 shows the distribution of participants regarding age, gender, hearing complaint, music listening habits and ototoxicity exposure in the groups studied. It shows that the predominant age group of the sound technicians was 25 to 35 years old (40.2%) and among the control group (54.7%), but without any significant statistical difference between age range frequencies (all age ranges presented p > 0.05) or between mean ages (p = 0.122). There was no statistically significant difference regarding the ratio of female professionals (p = 0.718) in the study groups. However, a significant statistical difference (p = 0.009 and p = 0.001) was observed between the ratio of hearing complaints and tinnitus, respectively, in the sound technician group and the ratio in the control group. The data suggest that there were a greater proportion of complaints in the sound technician group (26.8%) than in the control group (11.6%). There was no statistically significant difference between the two study groups in relation to music listening habits and ototoxicity exposure.

**Table 1:** Distribution of participants in the study groups according to age, gender, hearing complaints, music listening habits, tinnitus and presence of ototoxicity exposure.

**Variables**	**Sound technicians**		**Controls**		**p**
			
	n = 82	%	n = 95	%	
**Age**					

< 25	8	9.8	9	9.5	0.949^a^
25 to 35	33	40.2	52	54.7	0.054^a^
36 to 45	23	28.0	21	22.1	0.316^a^
> 45	18	22.0	13	13.7	0.149^a^
Mean ± SD	37.38 ± 9.98		35.09 ± 9.56		0.122^b^
Min-max	20–65		22–65		

**Gender**					

Female	4	4.9	4	4.2	0.718^a^
Male	78	95.1	91	95.8	

**Hearing Complaint**					

No	60	73.2	84	88.4	
Yes	22	26.8	11	11.6	0.009^a^

**Music listening habits**					

No	57	69.5	70	73.7	0.539^a^
Yes	25	30.5	25	26.3	

**Tinnitus**					

No	52	63.4	84	88.4	< 0.001^a^
Yes	30	36.6	11	11.6	
	
**Ototoxicity exposure**					
	
No	72	87.8	84	88.4	> 0.05
Yes	10	12.0	11	11.6	

^a^Chi-square test ^i ^Student's t test for homogenous independent samples

Table 2 shows the distribution of the participants in the sound technician group, according to the hours worked per day, length of time working as a professional and the use of hearing protection devices. It can be seen that the sound professionals predominantly worked for 6 to 12 hours per day (57.3%). Most of them had been working for between 5 and 14.9 years. However, most of them were not using hearing protectors (85.4%).

**Table 2:** Characterization of subjects according to the hours worked per day, length of time working and use of hearing protectors.

	**Distribution of participants of the sound professionals group**	
**Variables**	Frequency	%

**Hours worked/day**		

< 6 hours	24	29.3
6 to 12 hours	47	57.3
> 12 hours	11	13.4

**Length of time working as sound technicians**		

5.0 to 14.9	39	47.6
15.0 to 24.9	30	36.6
25.0 to 34.9	11	13.4
35.0 to 45.0	2	2.4
Average ± SD	15.9 ± 8.8	

**Use of hearing protectors**		

No	70	85.4
Yes	12	14.6

Table 3 shows the distribution of participants according to the presence of hearing loss in both study groups. A statistically significant difference between the sound technician group (50%) and control group (10.5%) can be seen in relation to high frequency hearing loss consistent with noise exposure, as demonstrated by the chi-square test (p < 0.001).

**Table 3:** Distribution of participants according to the presence of hearing loss.

**Variable**	**Sound technicians**		**Controls**		**p**
			
**Presence of hearing loss**	N = 82	%	N = 95	%	
No	41	50.0	85	89.5	<0.001^a^
Yes	41	50.0	10	10.5	

^a^Chi-square test

## Discussion

Workers exposed to noise levels of 85 dB have a permissible maximum daily average exposure of only eight hours and workers exposed to noise levels of 103 dB(A) have a permissible maximum daily average exposure of only 7.5 minutes [18]. However, most of the participants in the sound technician group at Cultura TV and at Record TV exceeded the sound level permitted for eight hours, given that 57.3% of the workers examined in this study were working for between 6 and 12 hours per day and 13.4% were working for more than 12 hours a day in a place proven to be inappropriate for hearing (Table 2), because the sound pressure level was above the permissible daily level. Most of the participants in the sound technician group said that they had been doing that type of work for between 5 and 14.9 years (47.6%) (Table 2). Studies on different populations exposed to sound have also demonstrated inappropriate mean sound levels of between 108 and 110 dB(A) [19-30].

We considered that individuals whose audiograms presented thresholds of less than or equal to 25 dB at all the frequencies examined presented normal hearing, in accordance with the standards of the Workplace SafetyOffice[4].

According to Santos & Morata (1999) [31], factors such as gender and age have been described as worsening factors for high frequency hearing loss consistent with noise exposure. However, in our study, these possible confounding factors were controlled for, because there was no statistically significant difference between the sound technician group and control group in these respects, thus rendering the study sample homogeneous without such bias in the results (Table 1). Furthermore, the individuals in both study groups were predominantly within the age range from 25 to 35 years old. Thus, young adults predominated in our study, with a consequently lower risk of developing presbyacusis (Table 1).

There was a statistically significant difference (p = 0.009) with regard to the presence of hearing complaints between the individuals in the sound technician and control groups (Table 1). Thus, the sound technicians had more hearing complaints than did the control group, and tinnitus was the most frequently reported complaint. These results were already expected and corroborate the results from various studies carried out among musicians in street carnival bands, disc jockeys, symphony orchestra musicians and others, which have described tinnitus and dizziness as the most frequently reported complaints [19-30].

There were no statistically significant differences in music listening habits and ototoxicity exposure between the groups studied (p > 0.05) (Table 1). However, the exposure to noise during leisure activities that was most mentioned in the studies that we researched in the literature and also in our study was going to discos and using a walkman [19-30].

Tinnitus is a symptom that may be found in workers suffering from high frequency hearing loss consistent with noise exposure and, according to Ferreira Júnior (1998) [32], such workers may mention that they have this symptom at times of hearing rest. It was found in our study that there was a statistically significant difference in the presence of tinnitus between the sound technician and control groups (p < 0.001), i.e. there was greater reporting of tinnitus among the sound technicians (Table 1).

One example of this situation of noise exposure over a long period of time is provided by the British guitar player and singer Eric Clapton (age 62 years), who developed tinnitus after spending years of his career playing in front of sound amplifiers at high volumes. He has said that, if he could go back in time, he would ask to have lower amplifier volume during his concerts. He has reported that he constantly hears "whistles" in his ears [33]. Another example that illustrates this situation within Brazilian popular music is provided by the vocalist of the group Jota Quest, Rogério Flausino, who is only 35 years old. In a documentary that forms part of the band's latest DVD, he reveals that he has lost 30% of his hearing because of many years living with high sound volumes [34]. Andrade et al (2000) [19] confirmed a possible association between the length of time working as a musician and the incidence of hearing alterations.

The main outcome of the present study was the presence of hearing loss. With regard to this, there was a statistically significant difference (p < 0.001) between the sound technicians and controls (50% versus 10.5%, respectively) (Table 3), thus revealing that hearing loss occurred approximately five times more frequently in the sound technician group. The difference could be addressed to high sound levels.

The effect of music on sound technicians' hearing was found to be greater in the present study than in other studies such as Cunningham (2006) [16]. This was perhaps because most of the sound technicians evaluated in the present study work or were working in open-air events, i.e. in places where the sound level is extremely higher and harmful.

Other study limitations might include residual confounding due to differences in socioeconomic status between the two study groups. This could partly explain the presence of much higher hearing loss levels among the sound technicians.

There is no effective treatment for permanent hearing loss resulting from excessive exposure to noise levels. However, this condition may generally be prevented through combined measures in programs against hearing loss (also called hearing conservation programs). Even though the efficacy of these measures (individual hearing protectors) for noise protection is determined by tests performed in laboratories, their effectiveness in preventing hearing loss due to exposure to cumulative noise depends mainly upon how regularly the workers use this protection. Studies have been demonstrating that if workers do not use hearing protectors for 100% of their working hours, the effectiveness of such devices will diminish rapidly. For example, using hearing protectors for only 90% of the time will diminish their effectiveness to less than one third of their efficacy [35].

85.4% of the participants in the sound technician group reported not using hearing protectors during their long working hours (Table 2). This seems to be a common phenomenon, because the majority of workers in manufacturing facilities, percussionists and musicians interviewed in other studies also reported not making use of hearing protectors. They were thus exposed to the risk of high frequency hearing loss consistent with noise exposure [16,36-39].

A systematic review has compared the effectiveness of intervention programs versus no intervention regarding the use of hearing protectors among workers exposed to noise levels above 85 dB(A). It was concluded that there was very little evidence regarding the efficacy of long-term school programs for increasing the use of hearing protectors among school students. Furthermore, the available evidence did not show that provision of customized/standardized information to motivate the workers to use hearing protectors was more effective than general information [40,41].

Programs for preventing hearing loss in industrial organizations have been widely defended. Occupational safety and health legislation obliges employees to have preventive measures in most countries [42]. In Europe and the USA, verification of noise exposure is mandatory, with periodic examinations on workers exposed to certain noise levels.

Studies in the USA have indicated that the use of hearing protectors is increasing, but there is still much to be improved in this respect [43]. Many factors have been reported as influencing the use of hearing protectors, such as the perception of risk likelihood and comfort when using the hearing protectors [35,44-46].

Implementation of hearing conservation programs within this population is of fundamental importance not only for the well being of these individuals, but also for the enrichment of Brazilian popular music, given that it is one of the best products of our country. The perception of hearing is related to the hearing threshold and maintenance of good perception through hearing protection measures will have the consequence of developing better professionals who can work to provide even greater value for Brazilian musicians.

For this to be definitively recognized and introduced within Brazilian culture and internationally, it will continue to be highly important, as a public health measure, to develop new strategies towards increasing the use of hearing protectors.

## Conclusion

(1) Sound technicians presented a higher prevalence of high frequency hearing loss consistent with noise exposure than did the general population, although the possibility of residual confounding by unmeasured factors such as socioeconomic status cannot be ruled out.

(2) There is some urgency with regard to carrying out studies aiming towards implementing a hearing conservation program through reducing sound levels and making sound technicians aware of the importance of using hearing protectors and adopting preventive actions.

## Competing interests

The authors declare that they have no competing interests.

## Authors' contributions

Conception and design: RED. Coordination of the study: VMT and EKS. Data acquisition: RED. Data analysis and interpretation: RED and JFM. Drafting the manuscript: RED. Critical revision for important intellectual content: RED. Data interpretation: RED and JFM.

## Pre-publication history

The pre-publication history for this paper can be accessed here:


